# Synthesis and Evaluation of Folate-Conjugated Phenanthraquinones for Tumor-Targeted Oxidative Chemotherapy

**DOI:** 10.4236/ojmc.2016.61001

**Published:** 2016-03-11

**Authors:** Ajay Kumar, Venkatesh Chelvam, Mahalingam Sakkarapalayam, Guo Li, Pedro Sanchez-Cruz, Natasha S. Piñero, Philip S. Low, Antonio E. Alegria

**Affiliations:** 1 International Center for Trans-disciplinary Research, School of Environmental Affairs, Universidad Metropolitana, San Juan, Puerto Rico; 2 Department of Chemistry, University of Puerto Rico, Humacao, Puerto Rico; 3 Department of Chemistry, Purdue University, West Lafayette, Indiana; 4 Department of Chemistry, Centre for Biosciences and Biomedical Engineering, Indian Institute of Technology, Indore, Simrol Campus, Madhya Pradesh, India

**Keywords:** Cancer, Folate Receptor, Reactive Oxygen Species

## Abstract

Almost all cells are easily killed by exposure to potent oxidants. Indeed, major pathogen defense mechanisms in both animal and plant kingdoms involve production of an oxidative burst, where host defense cells show an invading pathogen with reactive oxygen species (ROS). Although cancer cells can be similarly killed by ROS, development of oxidant-producing chemotherapies has been limited by their inherent nonspecificity and potential toxicity to healthy cells. In this paper, we describe the targeting of an ROS-generating molecule selectively to tumor cells using folate as the tumor-targeting ligand. For this purpose, we exploit the ability of 9,10-phenanthraquinone (PHQ) to enhance the continuous generation of H_2_O_2_ in the presence of ascorbic acid to establish a constitutive source of ROS within the tumor mass. We report here that incubation of folate receptor-expressing KB cells in culture with folate-PHQ plus ascorbate results in the death of the cancer cells with an IC_50_ of ~10 nM (folate-PHQ). We also demonstrate that a cleavable spacer linking folate to PHQ is significantly inferior to a noncleavable spacer, in contrast to most other folate-targeted therapeutic agents. Unfortunately, no evidence for folate-PHQ mediated tumor regression in murine tumor models is obtained, suggesting that unanticipated impediments to generation of cytotoxic quantities of ROS *in vivo* are encountered. Possible mechanisms and potential solutions to these unanticipated results are offered.

## 1. Introduction

Because cancer cells are rapidly mutating, they can develop resistance to almost any chemotherapeutic agent, resulting in recurrence of malignant disease. Thus, drug resistance can arise from mutations in the binding site of the drug on its therapeutic target, upregulation of multidrug resistance pumps, induction of immunosuppressive mechanisms, over-expression of the drug target, upregulation of catabolic pathways for drug degradation, or over-expression of alternate signaling pathways [1]. To avoid emergence of such resistance mechanisms, novel therapeutic agents will have to be designed that are immune to the above classes of escape mutations.

One class of drugs that could conceivably avoid escape mutations constitutes those molecules that continuously generate reactive oxygen species (ROS). Evidence that constant exposure to ROS does indeed constitute a mutation resistant cytotoxic mechanism derives from the fact that both the animal and plant kingdoms have exploited this strategy to kill invading pathogens since early evolutionary times [2] [3]. Indeed, oxidants and bleaches are still used in hospitals to sanitize areas exposed to potentially hazardous human body fluids [3]. Moreover, ROS generators need not enter cells to kill their targets and are consequently not subject to inactivation by multidrug resistance pumps. Although upregulation of antioxidant activity can confer some ROS resistance on certain cell types, cancer cells have been reported to have inherently poor antioxidant protection, and therefore, would be expected to be susceptible to killing by sustained oxidative stress [4]-[7].

Because autoxidation of ascorbate produces H_2_O_2_ as a final product [8] [9], ascorbate has been frequently proposed as a possible anticancer agent. Indeed, several recent reports have revisited the old controversy regarding the anticancer activity of ascorbate [10] [11] and have found significant cytotoxicity towards such cancers as prostate cancer [12]-[15], neuroblastoma [16], bladder carcinoma [15] [17], malignant mesothelioma [18] [19], chronic lymphocytic leukemia [20], hepatocellular carcinoma [15], mammary carcinoma [15] and cancer of the cervix [15]. In these studies, intravenous, but not oral, administration of ascorbate has been shown to generate both ascorbate radical and hydrogen peroxide in the extravascular space of both normal tissues [8] [21] and tumor xenografts [21]. These conditions not only result in decreased growth of tumor implants in mice [14] [15] [19] [22]-[26], but also enhanced toxicity when administered together with menadione (**1**), a redox-cycling quinone that can continuously generate ROS in the presence of ascorbate [22] [27] [28]. The rationale for supplementing ascorbate therapy with **1** is that quinones such as **1** catalyze ascorbate-mediated production of H_2_O_2_ [9] by first accepting an electron from ascorbate to form a semiquinone and then reacting with oxygen to generate superoxide anion radical, O_2_^•−^. The instability of this superoxide anion radical then results in its rapid disproportionation in water to hydrogen peroxide [29], which in turn can react via an iron-catalyzed Haber-Weiss reaction to form the hydroxyl radical [30]. Because of the nonspecific reactivity of the hydroxyl radical, it can damage a variety of biological systems via induction of lipid and protein peroxidation [31], degradation of deoxyribose [32] and promotion of DNA strand breaks [33] [34], leading to cell death.

There is evidence that the rate limiting step in O_2_^•−^ formation during redox cycling of simple quinones lies in the rate of quinone reduction and not reaction with oxygen [35]-[37]. Thus, rates of oxygen consumption by quinones in the presence of reducing agents are found to increase with quinone redox potential within a certain range of redox potentials, in accordance with Marcus electron transfer theory [35] [38]. We, therefore, hypothesized that if a more redox-active quinone than **1** was used in combination with ascorbate, more efficient anti-cancer activity should be observed.

Unfortunately, a major drawback of most oxidant-generating drugs is their lack of selectivity for malignant cells. Thus, when administered systemically, like other chemotherapeutic agents they distribute indiscriminately into all tissues, causing toxicity to malignant cells and healthy cells alike. For example, the LD_50_ value of **1** is 75 mg/rat Kg when administered intraperitoneally [39]. To circumvent this problem, we have exploited the use of folic acid to target attached drugs to folate receptors (FR) that are often over-expressed on cancer cells. Thus, human cancers known to upregulate FR relative to normal tissues include cancers of the ovary, lung, breast, kidney, brain, endometrium, colon, and hematopoietic cells of myelogenous origin [40]

Following binding to the FR, folate-drug conjugates are internalized by receptor-mediated endocytosis [41] and trafficked into recycling endosomes, where disulfide linkers connecting folate to its therapeutic payload can be cleaved, releasing the cytotoxic drug [42]. When desired, noncleavable linkers can also be designed that retain the drug in the targeting conjugate, preventing its release and diffusion from the cell. Folic acid (FA) conjugates of proteins, nucleotides, radiopharmaceuticals, imaging compounds, chemotherapeutic agents and a variety of nanoparticles have all been prepared and tested [43]-[46]. In most cases, FA conjugation to cytotoxic drugs has been shown to decrease their unwanted side effects and/or improve drug efficacy [43] [44].

In this paper, we have exploited folic acid to target redox cycling derivatives of 9,10-phenanthraquinone (PHQ) to cancer cells in an attempt to facilitate ascorbate-mediated oxidation and killing of malignant cells. We selected PHQ as the redox cycling catalyst because its more positive redox potential was found to facilitate oxidation of ascorbate ~20 times faster than menadione [9]. Moreover, unlike menadione, PHQ does not undergo Michael addition with free thiols, resulting in improved stability *in vivo* where free thiols can be common. In addition, we have recently observed that PHQ is only 14% less active in producing OH radicals from H_2_O_2_ + ascorbate than the classic Fenton reagent cation, Fe^2+^, at the same concentration (unpublished results). Thus, hydroxyl radical could also be produced where H_2_O_2_, ascorbate and PHQ are all coexisting.

## 2. Materials and Methods

### 2.1. Chemicals

Protected amino acids were purchased from AAPTEC or CHEMIMPEX.*N*^10^-(trifluoroacetyl)pteroic acid was purchased from Schirks. Other reagents are available from Sigma-Aldrich Chemicals, unless otherwise stated. Some of the important reagents are such as 3-acetylphenanthrene, mono-Boc-1,2-ethyldiamine. HCl, MeO-CO(O)SCl, 2-mercaptopyridine, Fmoc-Lys(mtt)-Wang resin, Fmoc-Cys(4-methoxytrityl)-Wang resin, N^10^TFA-pteroic acid, *δ*-protected Fmoc-Glu(O^t^Bu)OH [where Fmoc = fluorenylmethyloxycarbonyl & ^t^Bu = tertiary-butyl], diisopropylethylamine (DIPEA), *α*-protected Fmoc-Glu(O^t^Bu)OH, TFA, benzotriazol-1-yl-oxytripyrrolidino-phosphonium hexafluorophosphate (PyBOP), isopropanol (i-PrOH), piperidine, triisopropyl silane (TIPS), ethanedithiol (EDT), hydrazine, dichloromethane (DCM), dimethyl formamide (DMF), and diethyl ether.

### 2.2. Equipment

^1^H and ^13^C NMR spectra were recorded using a Bruker 400 MHz NMR spectrometer. LC-ESI-MS spectra were obtained using a Micromass Quattro Micro API triple quadrupole mass spectrometer with an ESI source. A Waters 1500 HPLC with a 20.0 mL loop injector was used for semi-preparative chromatography. An Agilent 1100 UV analytical HPLC chromatograph was used to identify compound purity.

### 2.3. Cell Culture and Animal Husbandry

KB, A-549 and MDA-MB-231 cells were obtained from American Type Culture Collection. MDA-MB-231 cells expressing high levels of FR were generated by passaging the cells for 14 weeks in folate-free cell culture medium. The cell lines were grown continuously as a monolayer in folate-free RPMI medium containing 10% fetal bovine serum and 1% penicillin/streptomycin antibiotic cocktail in a 5% CO_2_: 95% air-humidified atmosphere at 37°C.

All animal procedures were approved by the Purdue Animal Care and Use Committee in accordance with NIH guidelines. Normal rodent diets were not used, since they contain excessive amounts of folic acid which elevates serum folate levels significantly above normal physiological concentrations. Rather, all animals were maintained on a folate deficient diet (Harlan Teklad laboratories, WI) for at least 3 weeks prior to each study to lower their serum folate levels into the physiological range. Control animals were also maintained on a folate deficient diet.

### 2.4. Synthesis

#### 2.4.1. 3-Carboxyphenanthrenequinone

This compound was synthesized and purified as described by Jacobsen *et al.* by CrO_3_ oxidation of 3-acetylphenanthrene [47].

**Scheme 1:**
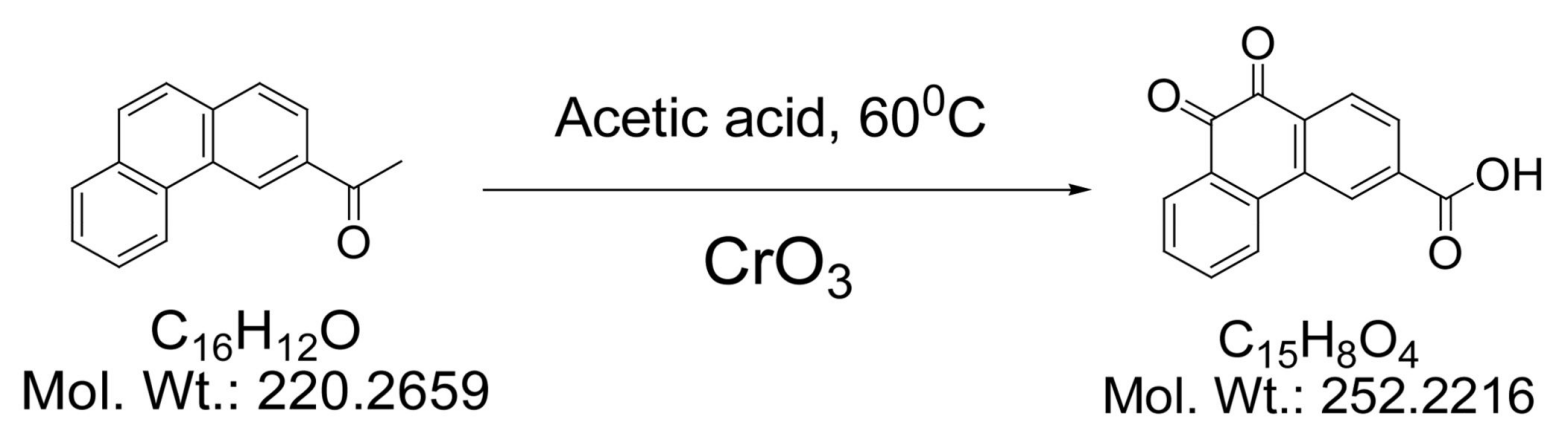
Synthesis of 3-carboxyllic phenanthrenequinone.

3-Carboxyllic phenanthrenequinone was synthesized and purified as described by Jacobsen *et al.* by CrO_3_ oxidation of 3-acetylphenanthrene.^60^ 3-Acetylphenanthrene (5 g, 0.23 mmol) was dissolved in warm acetic acid (100 ml, 60°C) and chrom(VI)oxide (30 g, 0.6 mol) was added in small portions (Scheme 1). During this procedure the temperature rose to the boiling point. After addition of all chrom(VI)oxide the solution was diluted with water (500 ml) and the yellow precipitate was filtered off, washed with acetic acid/H_2_O (1:1), cold acetic acid and finally with diethyl ether. Yield: 2.1 g (37% based on 3-acetylphenanthrene); MP: 280°C ^1^H NMR (400 MHz, DMSO-*d*_6_) *δ*: 7.577 (t, 1H, *J* = 7.55 Hz), 7.808 (t, 1H, *J* = 7.55 Hz), 8.041 (m, 2H), 8.120 (d, 1H, *J* = 8.12 Hz), 8.339 (d, 1H, *J* = 8.08 Hz), 8.706 (s, 1H), 13.636 (bs, 1H). ^13^C NMR (100 MHz, DMSO-*d*_6_) : ppm 125.00, 125.27, 129.64, 129.80, 129.92, 130.16, 132.00, 134.56, 134.94, 135.86, 135.95,136.74, 178.81, 178.87. ESI-MS: m/z 251 (calcd.) 252 (M + H)^+^ found.

#### 2.4.2. Non-Releasable FA-Conjugate of PHQ (3)

**Scheme 2:**
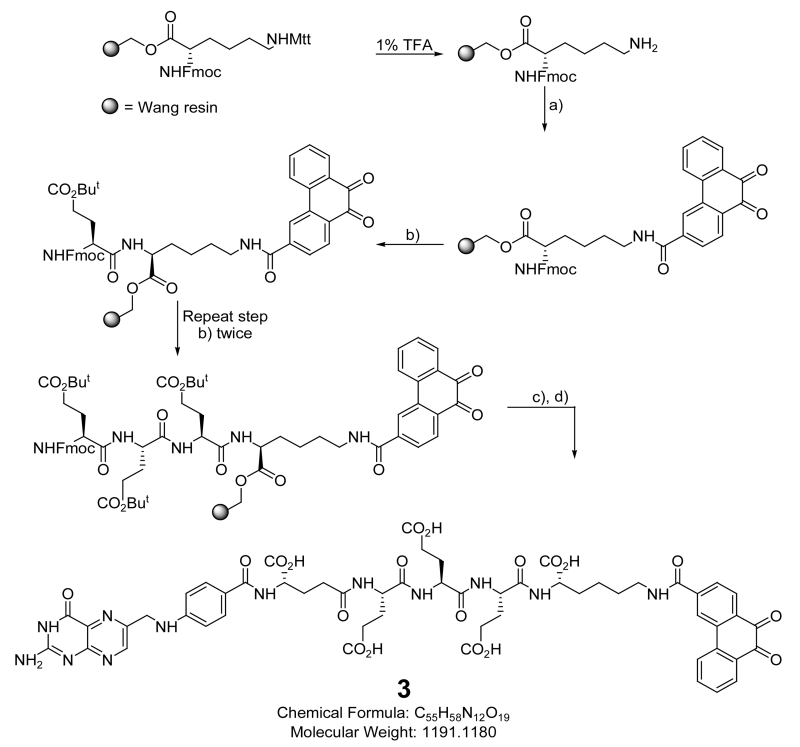
Synthesis of 3: reagents and conditions: (a) 3-carboxy-9,10-phenanthraquinone, PyBOP, DIPEA, DMF; (b) (i) 20% piperidine, DMF, (ii) Fmoc-Glu(OtBu)-OH, PyBOP, DIPEA, DMF; (c) (i) 20% piperidine, DMF, (ii) *N*(10)-TFA-pteroic acid, PyBOP, DIPEA, DMF; d) (i) TFA:TIPS:water (95:2.5:2.5:), (ii) sat.Na_2_CO_3_, pH = 10 - 11, 30 min.

The synthetic protocol for 3 appears in Scheme 2. The use of a lysine-containing resin enabled the coupling of both the quinone and folate-linker to the resin followed by detachment and purification.

1. Fmoc-Lys(mtt)-Wang resin (1.0 equiv) was swelled with DCM (3 mL) using a solid phase peptide synthesis vessel. After decanting, the swelling procedure was repeated with DMF (3 mL). After decanting DMF, 3 mL of 1% TFA in DMF was added to the resin and bubbled with Ar to promote homogeneous mixing for 5 min. This procedure was repeated 3 times and the resin was then washed with DMF (3 × 3 mL) and i-PrOH (3 × 3 mL). Formation of free amine was assessed by the Kaiser/ninhydrin test, where blue color shows the de-protection of amine and no more absorption at 304 nm in the de-protection waste showed complete deprotection.
2. The resin was swelled again in DMF. A solution of 3-carboxyllic phenanthraquinone (2.5 equiv), PyBOP (2.5 equiv), and DIPEA (4.0 equiv) in DMF was added and Ar was bubbled for 2 h. The resin was then washed with DMF (3 × 3 ml) and i-PrOH (3 × 3 mL). The coupling efficiency was assessed using the Kaiser test (absence of blue color indicates complete loading of protected glutamic acid).
3. The resin was swelled again in DMF and the solvent decanted. Three mLs of 20% piperidine in DMF was added to the resin and Ar bubbled for 5 min. This de-protection procedure was repeated three times and the resin was washed with DMF (3 × 3 mL) and i-PrOH (3 × 3 mL). Free amine formation was assessed by the Kaiser test.
4. The resin was again swelled in DMF and a solution of δ-protected Fmoc-Glu(O^t^Bu)-OH (2.5 equiv), PyBOP (2.5 equiv), and DIPEA (4.0 equiv) in DMF was added. Ar was bubbled for 2 h. The resin was then washed with DMF (3 × 3 ml) and i-PrOH (3 × 3 mL). The coupling efficiency was assessed using the Kaiser Test. Steps 3 and 4 were repeated to load two units of δ-protected Fmoc-Glu(O^t^Bu)-OH.
5. The resin was swelled in DMF and the solvent decanted. Three mLs of 20% piperidine in DMF were then added to the resin and Ar bubbled for 5 min. This procedure was repeated three times and the resin was washed with DMF (3 × 3 mL) and i-PrOH (3 × 3 mL). Formation of free amine was assessed by the Kaiser Test.
6. The resin was again swelled in DMF and a solution of α-protected Fmoc-Glu(O^t^Bu)-OH (2.5 equiv), PyBOP (2.5 equiv), and DIPEA (4.0 equiv) in DMF was added. Ar was bubbled for 2 h. The resin was then washed with DMF (3 × 3 ml) and i-PrOH (3 × 3 mL). The coupling efficiency was assessed using the Kaiser Test.
7. The resin was again swelled in DMF. A solution of *N*^10^-TFA-pteroic acid (1.25 equiv), PyBOP (2.5 equiv), and DIPEA (4.0 equiv) in DMF (and or DMSO) was then added. Ar was then bubbled for 8 h and the resin washed with DMF (3 × 3 ml) and i-PrOH (3 × 3 mL). The coupling efficiency was assessed using the Kaiser Test.
8. The resin was then washed with DMF (3 × 3 mL) and i-PrOH (3 × 3 mL). The final compound was cleaved from the resin using 3mL TFA:H_2_O:TIPS (95:2.5:2.5) cocktail (30 min × 3) and concentrated under vacuum. The concentrated product (**3**) was then precipitated in ice cold diethyl ether, centrifuged and dried under vacuum.
9. The crude product (**3**) was dispersed in deoxygenated Millipore water (5 mL) and the solution pH was adjusted to 10 - 11 using a deoxygenated saturated aqueous solution of Na_2_CO_3_ with continuous bubbling of argon through the solution for 30-min to de-protect *N*^10^-TFA on the folate moiety.
10. The crude product was purified by preparative RP-HPLC using a Waters XTerra MS C18 HPLC column 19 × 250 mm, 5 um, with detection at *λ* = 285 nm using the gradient 1%B to 50%B for 30 min, and washing with 80% B, for 5 min, where A = ammonium acetate buffer 20 mM, pH = 7 and B = ACN. Fractions were analyzed using analytical RP-HPLC and LC/MS. The combined fractions containing pure **3** were rotoevaporated under vacuum to remove ACN and lyophilized for 36 h to yield the final product as yellow solid. LC-MS: m/z 1190.4 (calcd.); found 1191.6 [M+H].

#### 2.4.3. Releasable FA-Conjugate of PHQ (4)

**Scheme 3:**
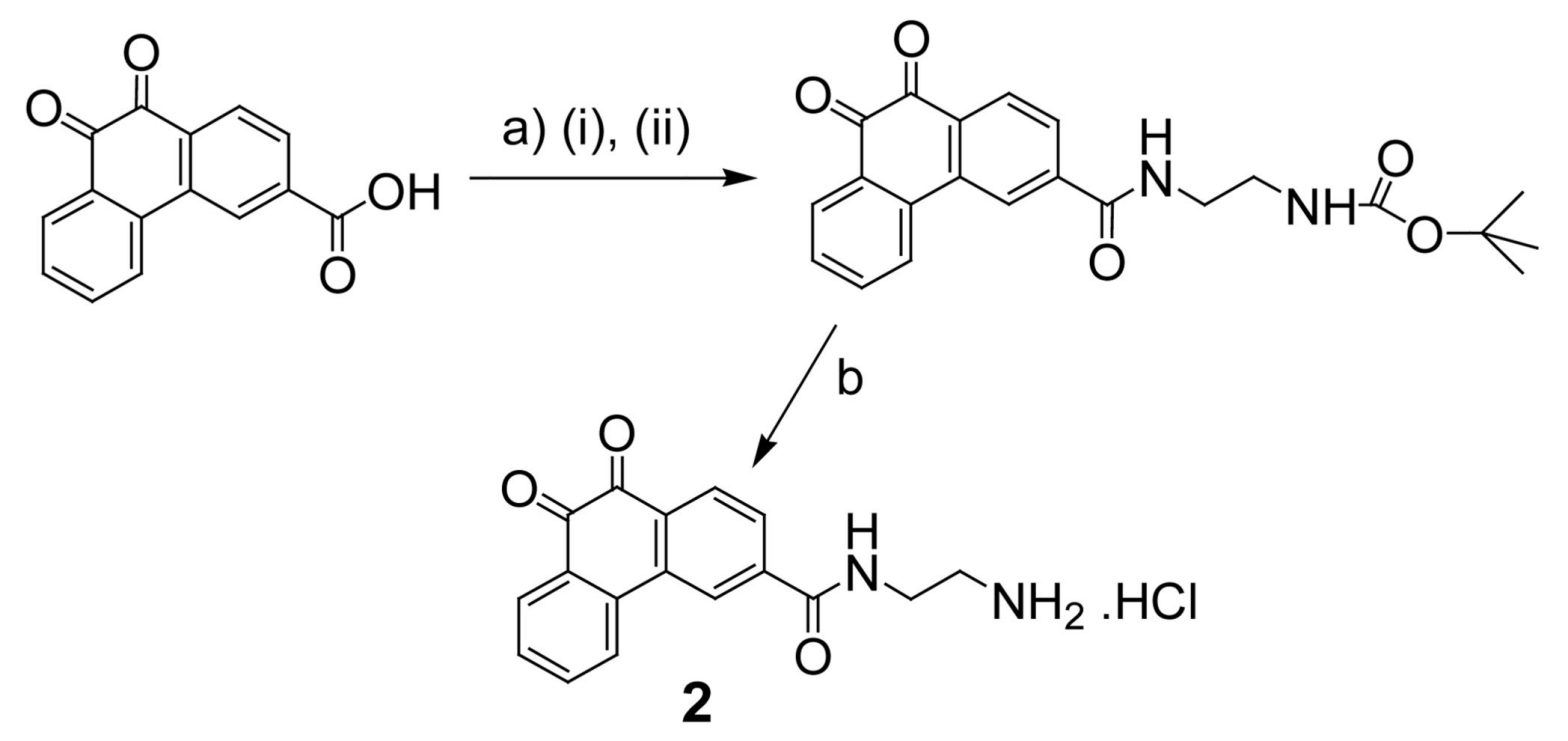
Synthesis of 2. Reagents and conditions: (a) (i) HOBt, DCC, dioxane, (ii) BocNH(CH_2_)_2_NH_2_, Et_3_N, CH_2_Cl_2_; (b) HCl, CH_3_COOH.

**Scheme 4:**
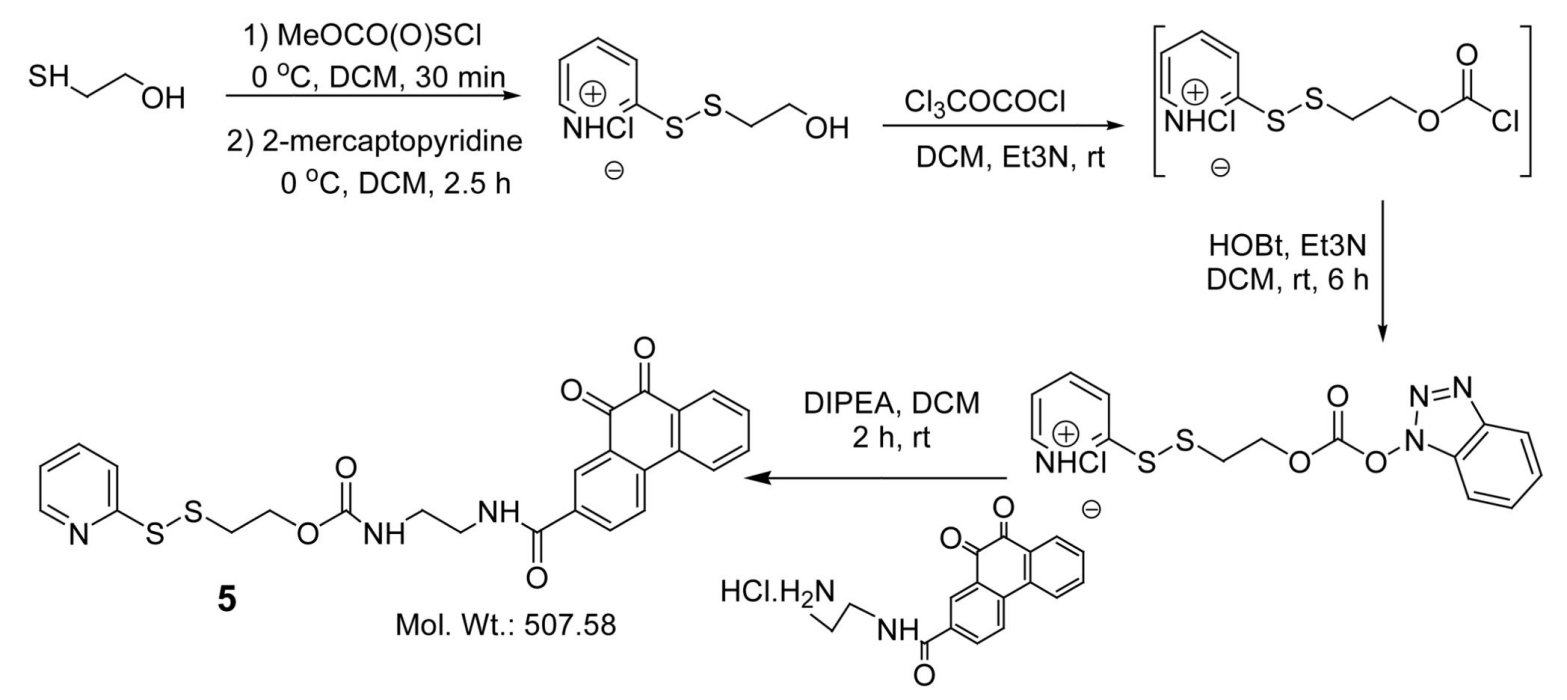
Synthesis of 5.

**Scheme 5:**
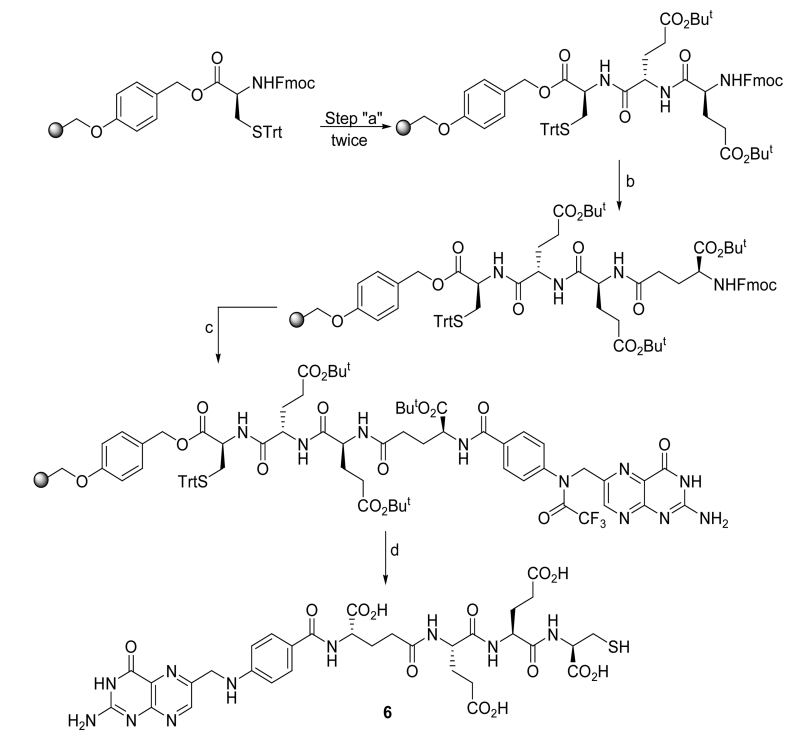
Synthesis of **6**: reagents and conditions: (a) (i) 20% piperidine/DMF, (ii) Fmoc-Glu(O^t^Bu)-OH, PyBOP, DIPEA/DMF; (b) (i) 20% piperidine/DMF, (ii) *α*-protected Fmoc-Glu(O^t^Bu)-OH, PyBOP, DIPEA/DMF; (c) (i) 20% piperidine/DMF, (ii) *N*^10^TFA-Pteroic acid, PyBOP, DIPEA/DMF; (d) (i) 2% NH_2_NH_2_/DMF, (ii) TFA:TIPS:Water:EDT (92.5:2.5:2.5:2.5).

**Scheme 6:**
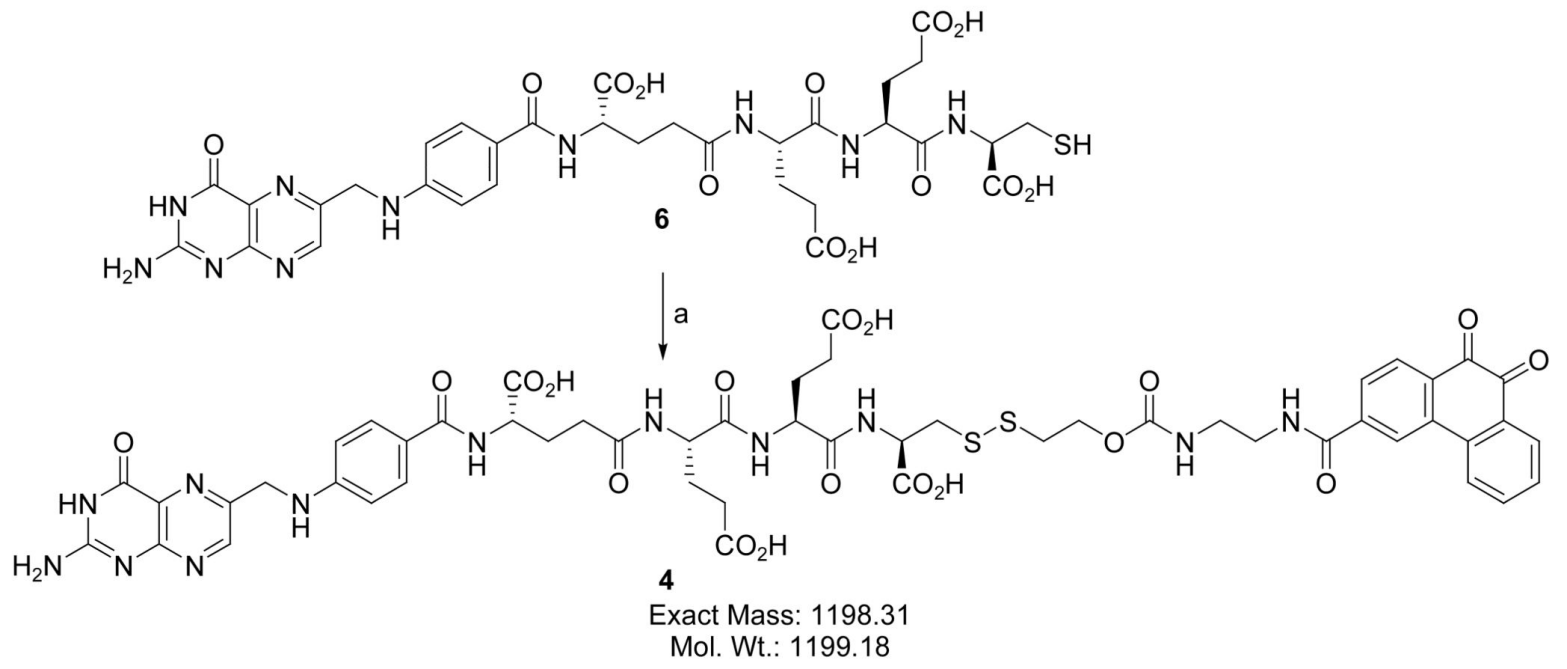
Synthesis of 4: (a) 5, THF:H_2_O (1:1), pH = 7.0, argon bubbling, rt, 45 min.

In order to synthesize this conjugate, several precursors were prepared. First, **2** and the pyridyldisulfide-substituted phenanthraquinone (**5**) were synthesized, Scheme 2. A folate-linker terminating with a cysteine (**6**) was also synthesized prior to assembly of the final product (**4**) (Schemes 3-6).

##### Synthesis of 2

###### Procedure

3-Carboxylic phenanthrenequinone (186 mg, 0.7 mmol), DCC (178 mg, 0.84 mmol), and HOBt (117 mg, 0.84 mmol) were dissolved in dry dioxane (50 ml), and the mixture was stirred overnight at room temp. The dioxane was evaporated in vacuo, and the residue was dissolved again in anhydrous DMF (25 ml). Mono-Boc-1,2-ethyldiamine.HCl ( 122 ul, 0.77 mmol) was added to the suspension/solution followed by excess triethylamine (1 ml). After 1 hour, DCU (dicyclohexyl urea) was filtered off and water was added (150 ml). The yellow precipitate was collected by filtration, washed several times with cold water. Yield: 230 mg (83.3%). De-protection of the amine: PHQ-3-EDABoc (47.4 mg) was dissolved slightly in warmed acetic acid (1.3ml, 50°C), and 1 M HCl in acetic acid was added (1.3 ml). After 5 minutes, cold ether was added (10 ml), and the precipitated 2 was collected by filtration and washed several times with ether followed by a single wash with acetonitrile. Yield: 39 mg (92%). ^1^H NMR (400 MHz, DMSO-*d*_6_) δ: 3.070 (bs, 2H), 3.584 (m, 2H), 7.590 (t, 1H, *J* = 7.52 Hz), 7.844 (t, 1H, *J* = 7.52 Hz), 7.959 (bm, 3H), 8.065 (d, 1H, *J* = 8.01 Hz), 8.111 (d, 1H, *J* = 8.01 Hz), 8.364 (d, 1H, *J* = 8.14 Hz), 8.678 (s, 1H), 9.083 (t, 1H, *J* = 5.32 Hz). ^13^C NMR (100 MHz, DMSO-*d*_6_): ppm 37.75, 39.02, 123.51, 124.98, 128.53, 129.58, 129.68, 130.14, 132.00, 133.51, 135.28, 135.73, 135.77, 139.92, 178.95, 179.02.

##### Synthesis of 5

Compound **5** was synthesized as described by Vlahov et.al.^61^ and purified by silica column using 10% EtOAc/Hexane. Yield 72%. ^1^H NMR (400 MHz, CDCl_3_) δ: 3.023 (t, 2H, *J* = 6.13 Hz), 3.523 (m, 2H), 3.634 (m, 2H), 4.385 (t, 2H, *J* = 6.09 Hz), 7.070 (m, 1H), 7.495 (bm, 1H), 7.513 (bm, 1H), 7.614 (bm, 1H), 7.733 (bm, 1H), 7.841 (d, 1H, *J* = 8.19 Hz), 8.186 (bm, 3H), 8.375 (d, 1H, *J* = 4.73 Hz), 8.563 (s, 1H). ^13^C NMR (100 MHz, CDCl_3_): ppm 37.758, 40.44, 42.28, 63.56, 119.93, 121.01, 123.56, 124.48, 127.41, 130.04, 130.66, 130.81, 131.13, 132.55, 135.32 136.29, 137.19, 140.55, 149.60, 157.99, 159.71, 170.00, 179.96,. LC/MS 507.58 (calcd.) 508.57 found [M+H]^+^.

##### Synthesis of 6

1. Fmoc-Cys(4-methoxytrityl)-Wang resin was swelled (1.0 equiv) with DCM (3 mL) using a solid phase peptide synthesis vessel. After decanting, the swelling procedure was repeated with DMF (3 mL). After decanting DMF, 3 mL of 20% piperidine in DMF was added to the resin and bubbled with Ar to promote homogeneous mixing for 5 min. This procedure was repeated 3 times and the resin was then washed with DMF (3 × 3 mL) and i-PrOH (3 × 3 mL). Formation of free amine was assessed by the Kaiser/ninhydrin test.
2. The resin was swelled again in DMF and the solvent decanted. Three mLs of 20% piperidine in DMF was added to the resin and Ar bubbled for 5 min. This procedure was repeated 3 times and the resin washed with DMF (3 × 3 mL) and i-PrOH (3 × 3 mL). Free amine formation was assessed by the Kaiser test.
3. The resin was again swelled in DMF and a solution of δ-protected Fmoc-Glu(O^t^Bu)-OH (2.5 equiv), PyBOP (2.5 equiv), and DIPEA (4.0 equiv) in DMF was added. Ar was bubbled for 2 h. The resin was then washed with DMF (3 × 3 ml) and i-PrOH (3 × 3 mL). The coupling efficiency was assessed using the Kaiser test. Steps 3 and 4 were repeated to load two units of δ-protected Fmoc-Glu(O^t^Bu)-OH.
4. The resin was swelled in DMF and the solvent decanted. Three mLs of 20% piperidine in DMF were then added to the resin and Ar bubbled for 5 min. This procedure was repeated three times and the resin washed with DMF (3 × 3 mL) and i-PrOH (3 × 3 mL). Free amine formation was assessed by the Kaiser Test.
5. The resin was again swelled in DMF and a solution of α-protected Fmoc-Glu(O^t^Bu)-OH (2.5 equiv), PyBOP (2.5 equiv), and DIPEA (4.0 equiv) in DMF was added. Ar was bubbled for 2 h. The resin was then washed with DMF (3 × 3 ml) and i-PrOH (3 × 3 mL). The coupling efficiency was assessed using the Kaiser Test.
6. The resin was again swelled in DMF. A solution of *N^10^*-TFA-pteroic acid (1.25 equiv), PyBOP (2.5 equiv), and DIPEA (4.0 equiv) in DMF was then added. Ar was then bubbled for 8 h and the resin washed with DMF (3 × 3 ml) and i-PrOH (3 × 3 mL). The coupling efficiency was assessed using the Kaiser Test.
7. The resin was then treated with 2% anhydrous hydrazine in DMF for 2-min (3 × 2 mL) and washed with DMF (3 × 3 mL) and i-PrOH (3 × 3 mL). The final compound was cleaved from the resin using 3mL TFA: H_2_O: TIPS (95:2.5:2.5) cocktail (30 min × 3) and concentrated under vacuum. The concentrated product **6** was then precipitated in ice old diethyl ether, centrifuged and dried under vacuum.
8. The crude product was purified using preparative RP-HPLC at λ = 285 nm (1%B to 50%B for 30 min, 80%B wash for 5 min; A = 0.1% TFA, pH = 2; B = ACN; column: Waters, xTerra C_18_ 10 μm; 19 × 250 mm, flow rate = 10 mL/min). Fractions were analyzed using analytical RP-HPLC and LC-MS: m/z 802 (calcd.) 803 found [M + H]. The fractions containing pure folate-Cys were combined, can was removed and lyophilized for 36 h to yield final product as yellow solid. LC-MS: m/z 802.2 (calcd.) 803.5 found [M + H]^+^.

##### Synthesis of 4

Compound **6** was dissolved in de-oxygenated H_2_O under argon bubbling with 0.1 N NaHCO_3_ resulting in a clear yellow solution at pH > 6.5. To this mixture it was added at once, under extensive stirring and bubbling of argon, a solution of **5** in THF. According to the HPLC profile, the reaction was completed in 20 min. HPLC purification gave pure conjugate **4**. LC-MS: m/z 1198.3 (calcd.) 1199.2 found [M + H]^+^.

### 2.5. Ascorbate Autoxidation

Oxygen consumption rate measurements were performed at 37.0°C ± 0.1°C in 20 mM phosphate buffer (pH 7.4). Stock and sample solutions were prepared with double distilled deionized water and decontaminated from traces of transition metals by exposure to Chelex 100 resin using the batch method [48]. Air-saturated sample solutions were used containing micromolar amounts of quinone and millimolar amounts of ascorbate (simulating parenteral ascorbate concentrations) and 20 mM phosphate buffer (pH 7.4). Oxygen consumption rates were measured with a 5300 Oxygen Biological Monitor (Yellow Springs Instruments Co., USA) using a Clark electrode as a sensor in a YSI 5301 constant temperature bath. Rates were calculated from the initial constant slopes of [O_2_] traces. Runs started in the absence of ascorbate followed by ascorbate addition, without interrupting oxygen consumption measurements.

### 2.6. Quinone Redox Potentials

**Figure 1:**
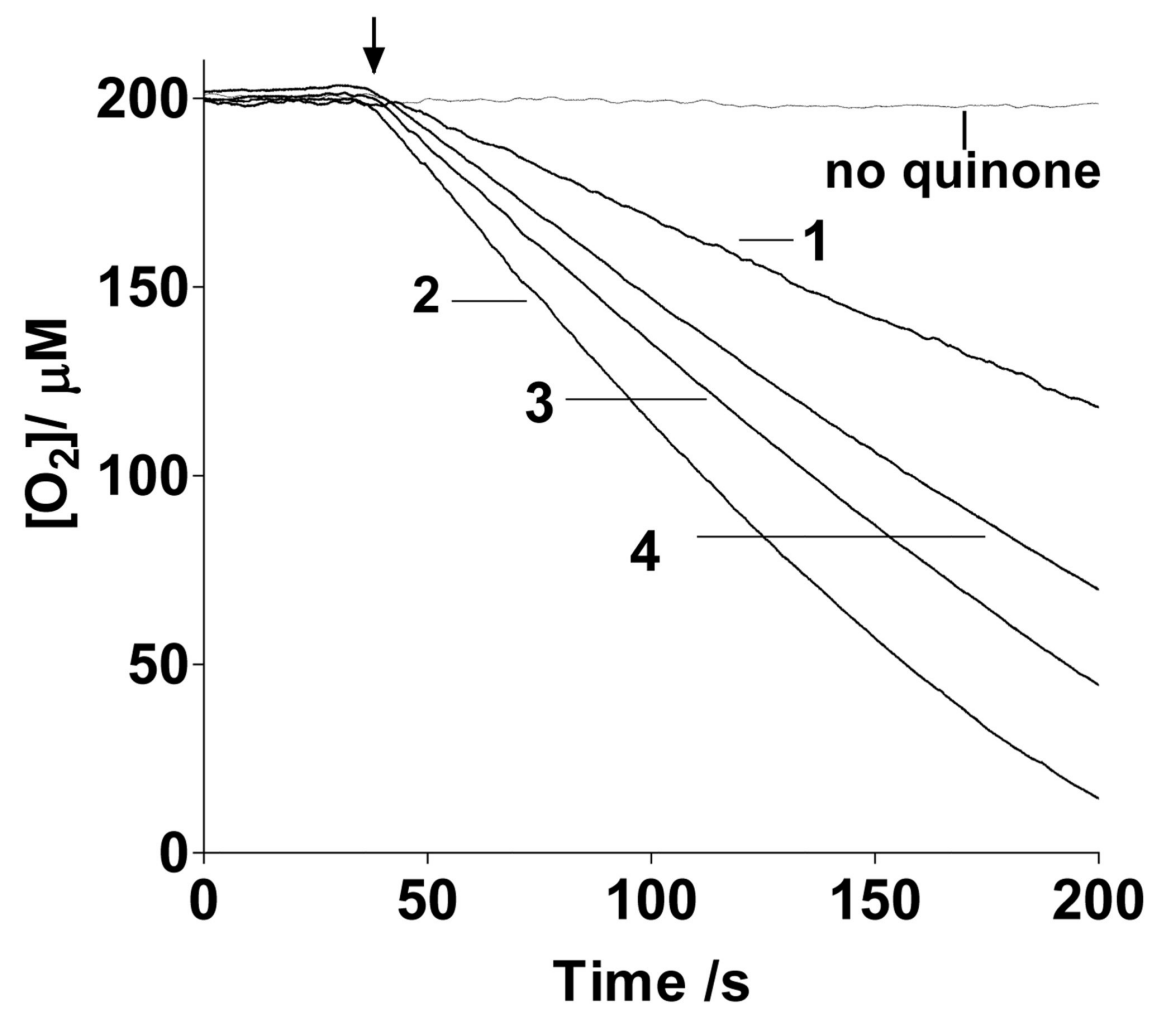
Oxygen consumption traces in air-saturated solutions containing 10 μM quinone and 1.00 mM ascorbate in 20 mM phosphate buffer at pH 7.4 and 37°C. The arrow indicates the time when ascorbate was injected. The numbers labeling the curves identify the quinone used to promote redox cycling.

Since **3** and **4** are not soluble in acetonitrile, half-wave reduction potentials (E_1/2_) were determined in 1:3 (v/v) nitrogen purged DMSO:20 mM phosphate buffer (pH 7.4). In this solvent a single peak corresponding to a 2 electron + 2 proton reduction step is observed for quinones [49]. Solutions contained up to 500 μM quinone and 0.1 M tetra-n-butylammonium perchlorate (TBAP). A BAS CV 50W voltammetric analyzer with a glassy carbon working electrode was used in these determinations. An Ag/AgCl(sat) electrode served as the reference electrode (E′ = +0.22 V vs. NHE) and a platinum wire as the counter electrode. Differential pulse voltammograms (DPV) were obtained in the potential range of −2.00 to 0.00 volts, using a 50 mV pulse amplitude and 20 mV/s of scan rate. Reduction potential values were obtained from the DPV peak potential maxima. These are very similar to the half-wave redox potentials, E_1/2_, in normal polarographic measurements [50]. Since compound **2** (see Figure 1 & Table 1) represents the quinone moiety existing in **3** and **4** and **1** has been used in previous quinone + ascorbate cytotoxicity and antitumor activity studies, redox potentials and ascorbate oxidation rates were also determined for these compounds.

### 2.7. *In Vitro* Cytotoxicity Assays of 2, 3 and 4

FR^+^KB cells and FR^−^ A-549 cells were seeded into 24-well (100,000 cells/well in 500 μL) Falcon plates and allowed to form monolayers over 12 h. Spent medium was replaced with fresh medium (0.5 mL) containing increasing concentrations of **2**, **3** or **4** and incubated for 2 h. The unbound test compounds **2**, **3** and **4** were washed from the wells with fresh medium (3 × 0.5 ml), followed by addition of 5, 3 and 0 mM (for a control experiments without sodium ascorbate) freshly prepared sodium ascorbate solution (pH = 7.4) in medium (0.5 mL/well). Cells were then incubated for an additional 2, 12, 16 or 68 h at 37°C. Cells were washed with fresh medium (3 × 0.5 ml) and incubated in fresh medium (0.5 mL) for 66, 56 or 0 h at 37°C. Spent medium in each well was replaced with fresh medium (0.5 mL) containing [^3^H]-thymidine (1 μCi/mL), and cells were incubated for 4 h at 37°C to allow [^3^H]-thymidine incorporation. Cells were then washed with medium (3 × 0.5 mL) and treated with 5% trichloroacetic acid (0.5 mL) for 10 min at room temperature. Cells were dissolved in 0.25 M NaOH (0.5 mL), transferred into individual vials containing Ecolume scintillation cocktail (3.0 mL), and counted in a scintillation counter (Packard, Packard Instrument Company). IC_50_ values were calculated by plotting % ^3^H-thymidine incorporation versus log concentration of compounds **2**, **3** and **4** using GraphPad Prism 4. Each % ^3^H-thymidine incorporation value represents the average ± standard error of the mean of at least three determinations.

### 2.8. Mouse Tumor Model

Four to five week-old female nu/nu mice were inoculated subcutaneously with KB or MDA-MB-231 cells (1.0 × 10^6^/mouse in cell growth medium) on their shoulders. Growth of the tumors was measured in two perpendicular directions every 2 days using a caliper (body weights were monitored on the same schedule), and the volumes of the tumors were calculated as 0.5 × *L* × *W*^2^ (*L* = longest axis and *W* = axis perpendicular to *L* in millimeters). Tumor therapy studies were performed with **3** (40 or 80 nmol/mouse) in saline (100 μL) 12 or 20 days after tumor cell implantation, when the tumors reached approximately 75 - 100 mm^3^ (for MDA-MB-231 tumor) and 100 - 350 mm^3^ (for KB-tumor) in volume.

### 2.9. *In Vivo* Potency of 3

Female nude mice bearing KB or MDA-MB-231 xenograft tumors were injected with **3** (2.0 μmol/kg for KB tumors of volume 100 - 350 mm^3^ or 4.0 μmol/kg for MDA-MB-231 tumors of volume 75 - 100 mm^3^) dissolved in 100 μL of saline via lateral tail vein injection. Treatments were conducted 3× per week for two weeks. A single dose of sodium ascorbate in saline (4g/kg or 80 mg/mouse) was injected 30-minutes post **3** injection and also on days when mice were not treated with **3.** Tumor volumes and body weights were measured on the same schedule. *In vivo* efficacy was evaluated by plotting tumor growth % versus days on therapy. Tumor growth % is defined by Equation (1),

(1) $$\text{Tumor Growth}\%=\left[\frac{V-V_{0}}{V_{0}}\right]\times 100\%$$

where *V* and *V*_0_ are the tumor volumes on any given day and the first day of measurement, respectively. Reported tumor growth percents are the averages ± standard errors of the mean of at least three determinations.

## 3. Results

### 3.1. Ascorbate Oxidation Rates *vs.* Redox Potentials

Upon addition of 1mM ascorbate to 10 μM quinone **1**, **2**, **3** or **4**, an increase in the rate of ascorbate oxidation is observed (Figure 1). The reason for including compounds **2** and **1** in this study is that the former mimics the PHQ moiety in the folate conjugate while the latter serves as an appropriate comparison to quinones used by others, since most of the anticancer work with quinones + ascorbate has been performed with **1**. It can be concluded from this figure that the ascorbate oxidation enhancement activity of these quinones follows the order **2** > **3** > **4** > **1,** which coincides with the ranking of the quinone 2 electrons+2 protons reduction potentials (Table 1).

### 3.2. Cytotoxicity Assays *in Vitro*

Quinone **2** displayed cytotoxic activity against KB cells at 3 and 5 mM ascorbate, with IC values in the 10^−8^ 50 M range, depending on incubation time (Table 2). Once this behavior was detected for 2, we were confident that a similar cytotoxic behavior was to be observed for **3** and **4** and thus were prompted to synthesize the FR-targeted quinones. Incubation of quinone **3** with 3 or 5 mM ascorbate for 2 hours exhibited very low cytotoxic activity *in vitro* (Table 2, entries 3 - 4), with the greatest cytotoxicity seen when cells were incubated for 12 hours (IC_50_ < 10^−9^ M) in the presence of either 3 or 5 mM ascorbate (Table 2, entries 6 - 7). In contrast, incubation of quinone **4** conjugate with 3 or 5 mM ascorbate for up to 68 hours was only slightly cytotoxic to KB cells (Table 2, entries 8 - 9) and no cytotoxicity was expressed when incubated for 12 hours with ascorbate. In addition, incubation of **3** in the absence of ascorbate with KB cells did not show cytotoxicity if incubated for 12 hours with ascorbate (Table 2, entry 5), indicating the need for ascorbate for cytotoxicity. Compound **3** in the presence of 3 mM ascorbate did not show toxicity, while it was mildly toxic when incubated for 12 hours, in the presence of 5 mM ascorbate, with FR negative A549 cells, thus demonstrating the need for cell surface binding to FR for expression of cytotoxic activity (Table 2, entries 10 - 11). The latter was expected, since **3** is extracted from FR negative cells upon rinsing due to its high hydrophilicity. These observations are in contrast to previous studies where KB [51] and A549 cells [21] were incubated for 1 to 2 hours with ascorbate followed by cell rinsing to remove free ascorbate and testing for cytotoxicity after incubation periods of more than one week. Ascorbate cytotoxicity to KB and A549 cells in the absence of quinones was then detected. Thus, interestingly, compound **3** is in fact accelerating the expression of this toxicity in FR+ cells.

### 3.3. Analysis of Ascorbate plus Folate-Quinone Conjugate Toxicity in Tumor-Bearing Mice

**Figure 2:**
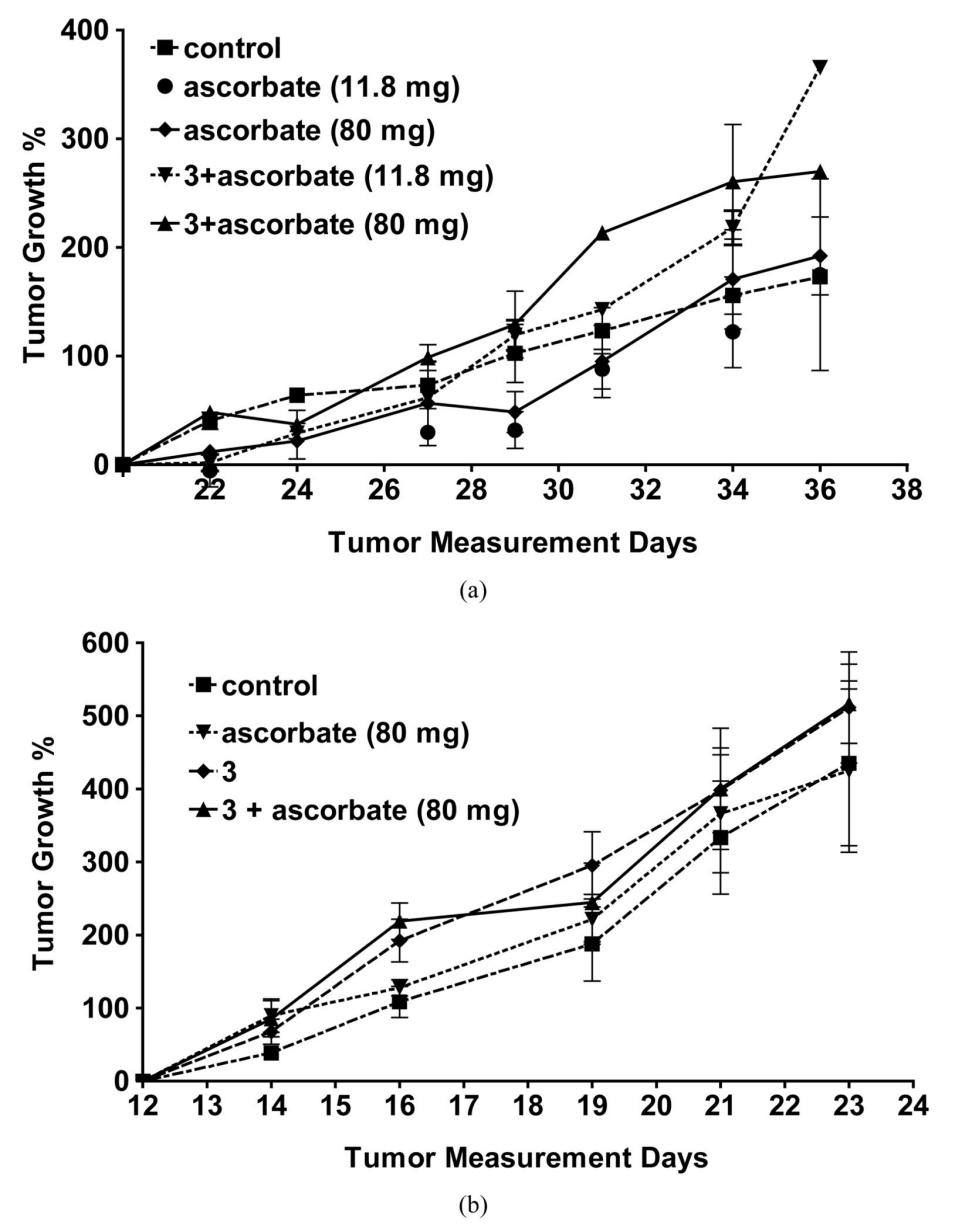
Effect of different combinations of ascorbate +/− various quinones on growth of FR+ tumor xenografts in athymic nude mice. (a) KB tumor implantation: 1 million KB cells were injected subcutaneously in 0.1 ml FD medium/mouse. Treatment began after the 20th day of tumor implantationwhen the average tumor size was 100 - 350 mm^3^. The controls are untreated mice. 3 was i.v. injected at a dose of 2.0 μmol/kg, 3 days/week for two weeks. Sodium ascorbate was injected intraperitoneally at a dose of 4 g/kg or 80 mg per mouse 30 min after i.v. injection of 3 or 11.8 mg per mouse every 30 min for 90-min, 30 min after i.v. injection of 3; (b) FR+ MDA-MB-231 tumor implantation: 1 million FR+ MDA-MB-231 cells were injected subcutaneously in 0.1 ml FD medium/mouse. Treatment began after the 12th day of tumor implantation when the average tumor size was 75 - 100 mm^3^. The controls are untreated mice. 3 was i.v. injected at a dose of 4.0 μmol/kg 3 days/week for two weeks. Sodium ascorbate was injected i.p. (4 g/kg or 80 mg/mouse) 30 min after 3 was injected and also on days when mice were not injected with 3. Tumor growth % values are averages ± standard errors of the mean at least four determinations.

Since the quinone conjugate **3** in combination with ascorbate demonstrated the greatest cytotoxicity *in vitro*, it was selected for more thorough testing against two FR+ tumor xenografts *in vivo*. As seen in Figure 2, using the protocol described in Materials and methods, essentially no reduction in tumor size was detected in either xenograft model, independent of whether quinone was injected or not. This result was not anticipated, since, as mentioned above, ascorbate alone, and ascorbate + **1**, have been reported to display some anti-tumor activity in murine tumor models, and because FR expression has been found to be similar in murine tumor xenografts and the cultured cancer cells from which they are derived (unpublished observations). Clearly, unknown factors dominate the behavior of ascorbate or folate-PHQ in our murine tumor models.

## 4. Discussion

### 4.1. Ascorbate Oxidation Rates *vs.* Redox Potentials

Although 2 electrons + 2 protons reduction potentials were measured here instead of 1 electron reduction potentials, the values still reflected the relative abilities of the quinones to undergo reduction, since previous work had shown that the rate of ascorbate oxidation in the presence of quinones increased with an increase in the one-electron redox potential of the quinone [9]. The electrochemical reaction, measured in the present work, Equation (2), corresponds to a redox potential (E_1/2_), which is essentially equal to the summation of the first and second electron redox potentials, Equation (3).

(2) $$2H^{+}+Q+2e\to {\mathrm{QH}}_{2}$$

(3) $$E_{1∕2}\approx E(Q∕Q^{•-})+E(Q^{?-}∕{\mathrm{QH}}_{2})$$

Thus, since in general a quinone with a more positive E(Q/Q^•−^) will also have a more positive E(Q^•−^/QH_2_) [52], the trend in E_1/2_ values shown here should roughly follow that in E(Q/Q^•−^) values. Not surprisingly, the relative order in E_1/2_ values correlates nicely with the rate of ascorbate oxidation in Table 1. The discrepancy in ascorbate oxidation rates and in reduction potentials among compounds 2, 3 and 4 is an interesting subject. First of all, at physiological pH, compound 2 should be mostly protonated at the primary amine moiety. Addition of the folate linkers to the quinone moiety not only will increase the steric hindrance upon approaching the ascorbate ion, but the amide group attached to the quinone moiety has a different pKa value than that of the amine group in compound **2**. Those characteristics may be responsible for the smaller redox reactivity of **3** and **4** as compared to **2**. Differences in redox reactivity between **3** and **4** may be due to differences in steric or structural conformations when approaching ascorbate, since the disulfide bond is not being reduced by ascorbate. However, the elucidation of the molecular causes for these redox reactivity differences is beyond the scope of this work, which is intended to explore the relative toxicities of releasable and non-releasable FR-targeted quinones.

### 4.2. Cytotoxicity Assays

Since ascorbate oxidation by quinones generates H_2_O_2_ and other ROS, it is expected that all PHQ derived compounds would show significant cytotoxic activity when combined with ascorbate. However, neither FA-bound **3** nor **4** showed cytotoxicity to KB cells when those compounds were incubate for only 2 hours in the presence of 3 or 5 mM ascorbate, nor compound **3**, the more potent of the two folate conjugates, required 12 h of incubation with ascorbate to achieve its full potency in the subpicomolar range. Although compound **4** exhibits limited toxicity to KB cells after 68 hours of incubation with ascorbate, no cytotoxicity was expressed when incubated with ascorbate for 12 hours.

Previous research on folate-targeted therapeutic agents has suggested that folate-linked cytotoxic warheads must be released from folate to achieve maximum cytotoxicity. To promote this release, self-immolative linkers, generally containing a disulfide bond in close proximity to a labile ester bridge, have been employed to attach folate to its cytotoxic cargo [42] [53]. However, in our studies, the non-releasable FA-bound **3** displayed significantly more potent activity than the releasable FA-bound **4**. Incubation of compound **4** with 5 mM ascorbate did not release the quinone even after 24 hours of incubation, as detected from HPLC sample analysis. Furthermore, a decrease in the HPLC peak of **4** was not detected either. Many studies have shown that exogenous dehydroascorbic acid is transported into cells where it is reduced to ascorbic acid by glutathione [54]. The latter is in line with the fact that the ascorbate to dehydroascorbate oxidation potential is +0.10 V, while the oxidation potential of GSH to GSSH is + 0.24 V [55], indicating that GSH, which is located inside the cell in relatively large concentrations, should efficiently reduce the disulfide moiety in **4**, as opposed to ascorbate. In fact, to our best knowledge, all the disulfide-containing folate-targeted drugs reported in the literature are known to release the drugs inside the cells, thus improving their cytotoxic activity as compared to non-releasable FR-targeted analogs [56].

The lack of toxicity of **4** might have been caused by glucuronide conjugation of the released phenanthraquinone moiety inside the cell, as previously observed for phenanthraquinone [57]. This will decrease the amount of released PHQ available for ROS production. Another cause for the lack of toxicity of **4** could be its smaller redox potential which correlates with its smaller rate of ascorbate oxidation (Figure 1, Table 1). FA-bound **3**, in contrast, would be expected to remain cell associated due to the high affinity of folate for FR [56], forcing the ROS generator to accompany FR wherever the receptor traffics within the cell and at the cell membrane.

### 4.3. Ascorbate and Folate Conjugate Administration to Tumor-Bearing Mice

No tumor size reduction or decrease in tumor growth rate was detected after i.v. injection of either ascorbate, ascorbate + conjugate **3**, or conjugate **3** alone. This observation contrasts with previous results seen following both i.p. administration of ascorbate alone as well as ascorbate + **1** mixture [27]. A possible explanation for the absence of anti-tumor activity *in vivo* may derive from the fact that neither KB nor MDA-MB-231 tumors are highly vascularized, and since ascorbate/quinone-mediated ROS production requires molecular O_2_, the pressure of O_2_ in the tissues could have been insufficient to enable adequate ROS generation in the tumors. Whether a more active quinone catalyst can be found that can compensate for the lower oxygen tensions that often exist in tumors will have to await further investigation.

A second possible explanation for the insensitivity of KB and MDA-MB-231 tumors to the ascorbate/quinone combination *in vivo* could have arisen from an inadequate concentration of the quinone moiety in the tumor. Thus, Verrax *et al.* reported antitumor activity of **1** + ascorbate after their i.p. injection in mice [27]. Although less ascorbate was injected in mice in their study (1 g/mouse Kg vs. 4 g/mouse Kg), 15 times more of **1** than the amount of **3** used in our work was injected. Furthermore, previous work from our lab has demonstrated that an average of ~2 μmol/Kg of FR-targeted compound will saturate KB tumors in mice, and any additional folate conjugate injected will simply be excreted [58]. This saturating concentration (*i.e.* ~2 μmol/Kg) is 30 times lower than the amount of **1** used by Verrax *et al.* Furthermore, FR-targeted compounds generally saturate all FR in KB xenografts within 5 minutes of tail injection with kinetics that are largely independent of the type of folate linker [56]. In previous work, ascorbate concentrations of up to 8 mM and H_2_O_2_ concentrations of up to 20 μM were detected in the extracellular fluid at 20 to 30 minutes after tail injection of 0.5 mg ascorbate/g mouse [8]. Furthermore, H_2_O_2_ concentrations ≥ 25 μM *in vitro* were found to be toxic to cancer cells [59]. In the current work we are injecting 8 times more ascorbate/g of mouse (4 mg/g). Thus, larger peak concentrations of ascorbate and even higher levels of H_2_O_2_ (due to the targeted PHQ) in the intracellular fluid must have been produced at the tumor tissues described in the current work. However, taken together the fact that **3** is water-soluble and **1** is hydrophobic, it is conceivable that **3** might be more rapidly excreted than **1**, while **1** might partition into cells more efficiently than **3**, rendering the amount of **3** available for ascorbate reduction at the tumor site insufficient to generate the cytotoxic levels of ROS induced by **1**. While differences in drug metabolism, tumor size, levels of ROS detoxifying enzymes, ascorbate excretion, etc. could have also contributed to the insensitivity of our murine tumor models to the ascorbate/quinone oxidant generating system*,* an unambiguous description of the actual mechanism is beyond the scope of this study.

In summary, no known eukaryotic cells can survive prolonged exposure to ROS, suggesting that constitutive generators of reactive oxygen species could constitute an evasion-proof method for killing essentially all tumor cells. In an effort to design such a mutation-resistant chemotherapy, the combination of a PHQ-derived non-releasable FR-targeted compound with ascorbate was investigated. Our data demonstrate a significant acceleration of the ascorbate cytotoxicity against FR+ KB cells, promoted by the presence of **3**, if **3** is incubated with ascorbate *in vitro*, but not *in vivo*. Since, as described above, related nontargeted therapies from other labs have shown activity against other types of implanted tumors, our observations suggest that FR-targeting of quinones to KB and MDA-MB-231 tumors may be impaired by either poor vascularization and/or a limited concentration of quinone at the tumor site as compared to nontargeted quinone-ascorbate-based therapies.

## Figures and Tables

**Table 1:** Ascorbate oxidation rates (Rox)^a^ and quinone redox potentials (E_1/2_)^b^.

Quinone	Rox/μM·s^−1^	E_1/2_/mV
2	1.30 ± 0.06	−304
3	1.02 ± 0.05	−316
4	0.87 ± 0.08	−336
1	0.54 ± 0.04	−372

a Obtained after addition of 1 mM ascorbate to solutions containing 10 μM quinone in 20 mM, air-saturated, phosphate buffer.

b Measured in 1:4 (v/v) DMSO:20 mM phosphate buffer (pH 7.4). DPV peak potential maxima are against Ag/AgCl.).

**Table 2:** Cytotoxicity to KB (FR^+^) and A549 (FR^−^) cells of compounds 2, 3, 4 after incubation for 2 h followed by washing off unbound compounds with fresh medium (3 × 0.5 mL) and different incubation time in the presence of 3 mM or 5 mM ascorbate solution.^b^

S. No.	Compound No.	Cell line	Ascorbate concentration (mM)	Incubation time of cells with ascorbate (h)	IC_50_ nM (Nanomolar), pM (Pico molar)
1.	2	KB (FR^+^)	3	16	14.7 nM
2.	2	KB (FR^+^)	5	16	12.6 nM
3.	3	KB (FR^+^)	3	2	Non-toxic
4.	3	KB (FR^+^)	5	2	Non-toxic
5.	3	KB (FR^+^)	0	12	Non-toxic
6.	3	KB (FR^+^)	3	12	0.14 pM
7.	3	KB (FR^+^)	5	12	0.0011 pM
8.	4	KB (FR^+^)	3	68	Non-toxic
9.	4	KB (FR^+^)	5	68	Non-toxic
10	3	A549 (FR^−^)	3	12	Non-toxic
11.	3	A549 (FR^−^)	5	12	Non-toxic
